# Induction of cytochrome P450 via upregulation of CAR and PXR: a potential mechanism for altered florfenicol metabolism by macranthoidin B *in vivo*

**DOI:** 10.3389/fphar.2024.1460948

**Published:** 2024-10-09

**Authors:** Si-cong Li, Bin Wang, Min Zhang, Qin Yin, Zi-yi Yang, Xu-ting Li, Ge Liang

**Affiliations:** ^1^ Animal Breeding and Genetics key Laboratory of Sichuan Province, Sichuan Animal Science Academy, Chengdu, China; ^2^ Veterinary Natural Medicine Research and Good Clinical Practice Experimental Animal Centre, Lezhi, China

**Keywords:** macranthoidin B, cytochrome P450, constitutive androstane receptor, pregnane X receptor, florfenicol, pharmacokinetics

## Abstract

**Introduction:**

Macranthoidin B (MB) is a primary active component of *Flos Lonicerae.* In Chinese veterinary clinics, *Flos Lonicerae* is frequently used in combination with florfenicol to prevent and treat infections in livestock and poultry. However, potential interactions between *Flos Lonicerae* and florfenicol remain unclear. To systematically study these interactions, it is crucial to investigate the individual phytochemicals within *Flos Lonicerae*. Therefore, MB was selected for this study to assess its effect on the pharmacokinetics of florfenicol *in vivo* and to explore the underlying mechanisms involved.

**Methods:**

Male Sprague-Dawley rats were administered MB (60 mg/kg BW) or sterile water orally for 7 consecutive days. On the 8th day, a single oral dose of florfenicol (25 mg/kg BW) was given. Florfenicol pharmacokinetics were analyzed using ultra-high performance liquid chromatography. The hepatic expression levels of cytochrome P450 (CYP1A2, CYP2C11, CYP3A1), UDP-glucuronosyltransferase (UGT1A1), P-glycoprotein (P-gp), and nuclear receptors, including constitutive androstane receptor (CAR), pregnane X receptor (PXR), and retinoid X receptor alpha (RXRα), were quantified via reverse transcription-quantitative polymerase chain reaction and Western blotting (WB). Hepatic CYP1A2 and CYP2C11 activities were measured using a cocktail method. Additionally, the subcellular expression and localization of CAR, PXR, and RXRαin hepatocytes was assessed using WB and immunofluorescence staining.

**Results:**

MB significantly reduces the AUC_(0-∞)_ and MRT_(0-∞)_ of florfenicol. MB also markedly upregulates the mRNA and protein expression of hepatic CYP1A2 and CYP2C11, along with their catalytic activities. Substantial upregulation of CAR and PXR proteins occurs in the hepatocyte nucleus, along with significant nuclear colocalization of the transcriptionally active CAR/RXRα and PXR/RXRαheterodimers, indicating MB-induced nuclear translocation of both CAR and PXR.

**Discussion:**

These findings suggest that MB-induced alterations in florfenicol pharmacokinetics, particularly its accelerated elimination, may be due to increased expression and activities of CYP1A2 and CYP2C11, with CAR and PXR potentially involved in these regulatory effects. Further investigation is yet needed to fully elucidate the clinical implications of these interactions concerning the efficacy of florfenicol in veterinary medicine.

## Introduction

The use of herbal medicines is a common practice in Chinese veterinary therapeutics. Herbal treatments are frequently combined with conventional drugs for disease prevention and control. However, the phytochemicals in these herbs can interact with co-administered drugs by either inducing or inhibiting drug-metabolizing enzymes and/or efflux transporters. This can influence drug metabolism and potentially create undesirable interactions (van Erp et al., 2005).

**FIGURE 1:**
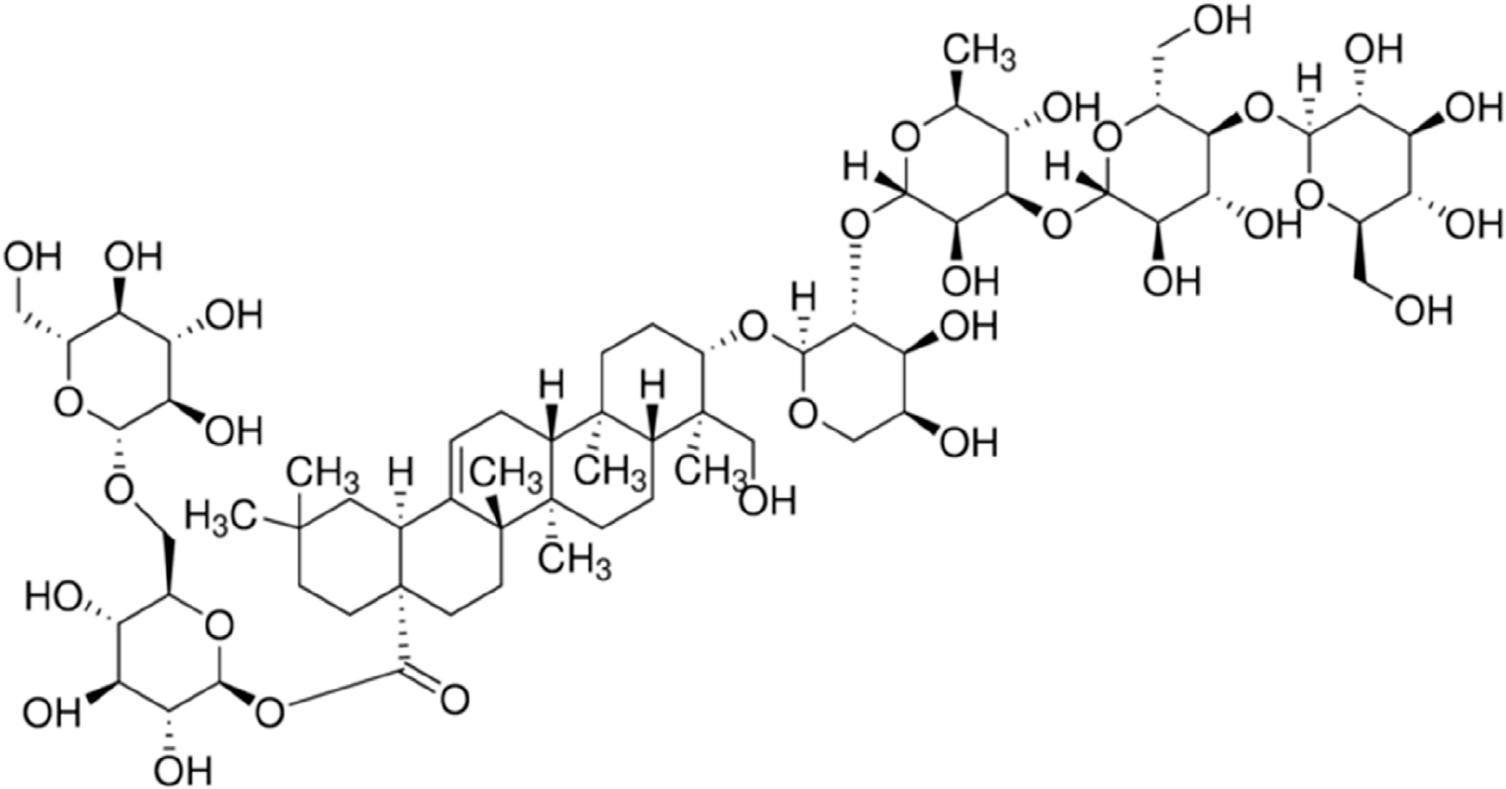
Chemical structure of macranthoidin B.

*Flos Lonicerae* (“Shanyinhua” in Chinese) is a traditional Chinese medicine listed in the 2020 edition of the Veterinary Pharmacopoeia of the People’s Republic of China. One of its primary active components is Macranthoidin B (MB), a triterpenoid compound constituting approximately 5%–10% of the total content of the herb (Figure 1) (Zhao et al., 2015; Zhou et al., 2023). MB is also listed in the Veterinary Pharmacopoeia as a standard substance for the quality control of *Flos Lonicerae*. *Flos Lonicerae* is regarded for its heat-clearing and detoxifying properties. It is frequently incorporated into herbal preparations for treating infectious diseases in livestock and poultry due to its antimicrobial and anti-inflammatory effects, and often combined with antibiotics to enhance therapeutic efficacy (Li C. Y. et al., 2023; Chinese Veterinary Pharmacopoeia Committee, 2020). Florfenicol, a veterinary-specific antibiotic, is characterized by excellent absorption, broad distribution within the body, and a wide spectrum of antibacterial activity; this drug is also commonly used to treat bacterial infections in livestock and poultry. Given the extensive use of both *Flos Lonicerae* and florfenicol in veterinary practice, it is crucial to assess potential interactions between these two compounds.

Efflux transporters and drug-metabolizing enzymes are key factors in herb-drug interactions, as they significantly influence drug metabolism. P-glycoprotein (P-gp), which is encoded by the multidrug resistance 1 (MDR1) gene, is an important efflux transporter playing a major role in drug clearance. P-gp is localized at the canalicular membrane of hepatocytes in the liver, where it aids in excreting drug metabolites into bile (Lagas et al., 2010; Elmeliegy et al., 2020). In rabbits and chickens, P-gp has been implicated in the metabolism of florfenicol (Liu et al., 2012; Wang et al., 2018). Phase I drug metabolism is primarily mediated by enzymes belonging to the cytochrome P450 (CYP) superfamily, which are crucial for drug biotransformation. In humans, key enzymes include CYP1A2, CYP2C9, and CYP3A4 (Zhou et al., 2009; Lee et al., 2013; Shi et al., 2021), with homologous enzymes in rats such as CYP1A2, CYP2C11, and CYP3A1 (Xu et al., 2014; Geng et al., 2015; Ramakrishna et al., 2016; Ma et al., 2019). CYP1A2 and CYP3A have been found to participate in the metabolism of florfenicol in rats and rabbits, respectively (Liu, 2011; Liu et al., 2012). Phase II metabolism primarily involves conjugation reactions mediated by UDP-glucuronosyltransferases (UGTs). UGT1A1 is a particularly significant enzyme involved in drug metabolism (Wen et al., 2007; Kapitanović et al., 2009; Yao et al., 2019; Zhu et al., 2021). Given the critical roles of these enzymes and transporters in the metabolism of various drugs, they are important subjects of herb-drug interaction research.

The constitutive androstane receptor (CAR) and pregnane X receptor (PXR), both part of the nuclear receptor superfamily, are principal regulators involved in the transcriptional control of phase I and phase II drug-metabolizing enzymes and efflux transporters. These include CYP450, UGT1A1, and P-gp (Moore et al., 2000; Tolson and Wang, 2010; Kanno et al., 2016; Buchman et al., 2018; Rakateli et al., 2023). Upon binding to agonists or ligands, conformational changes occur in their ligand-binding domains, causing corepressors to dissociate and coactivators to be recruited. This process culminates in the formation of active heterodimers with retinoid X receptor alpha (RXRα), another nuclear receptor, facilitating nuclear translocation and subsequent activation of target gene transcription (Mangelsdorf and Evans, 1995; Hashimoto and Miyachi, 2005; Wang et al., 2019). Consequently, this regulatory pathway can alter the pharmacokinetics of substrates for these enzymes and transporters.

Studying the interaction mechanisms between *Flos Lonicerae* and florfenicol requires a systematic approach, beginning with the investigation of individual phytochemicals within *Flos Lonicerae*. In this study, we selected MB, one of the major compounds in *Flos Lonicerae*, as a starting point. We first assessed how MB affects florfenicol pharmacokinetics, followed by an examination of its influence on the expression of key liver enzymes, including CYP1A2, CYP2C11, CYP3A1, and UGT1A1, as well as the efflux transporter P-gp. Additionally, we explored how MB impacts the expression and localization of nuclear receptors, such as CAR, PXR, and RXRα, in hepatocytes. These findings may improve our understanding of MB’s role in florfenicol metabolism and provide a foundation for future studies on the molecular mechanisms underlying interactions between *Flos Lonicerae* and florfenicol. Ultimately, this research may offer a scientific basis for the integrated use of herbal and conventional drugs in veterinary medicine.

## Materials and methods

### Chemicals, reagents

The chemical compounds used in this study were obtained from reputable suppliers. MB (purity 96.8%; CAS No. 136849-88-2) was sourced from Chengdu Desite Biotechnology Co., Ltd. (Chengdu, China). Florfenicol was provided by Hubei Longxiang Pharmaceutical Tech. Co., Ltd. (Huanggang, China). Prior to experimentation, it was dissolved in polyethylene glycol 400 (Kelilong, Chengdu, China) at a 25 mg/mL concentration. The florfenicol analytical standard was acquired from the China Institute of Veterinary Drug Control (content 99.1%). Analytical standards of chloramphenicol (internal standard; content 99.8%) and carbamazepine (internal standard; content 99.9%) were obtained from the National Institutes for Food and Drug Control (Beijing, China), and phenacetin (purity > 98%) and tolbutamide (purity > 98%) from Sigma (St. Louis, MO, United States). A mixed suspension of phenacetin (4 mg/mL) and tolbutamide (2 mg/mL) was freshly prepared in a 0.2% carboxymethylcellulose sodium solution immediately before use. Acetonitrile, methanol, and ethyl acetate of HPLC grade were sourced from Merck Chemicals Co., Ltd (Darmstadt, Germany). All other chemicals were of analytical grade. HPLC-grade water was prepared using a Milli-Q system (Millipore, Bedford, United States).

The SYBR Green PCR Kit (QIAGEN, Frankfurt, Germany) was used for mRNA detection. Rorward and reverse primers were obtained from Sangon Biotech Co., Ltd (Shanghai, China). TRIzol reagent and a RevertAid First Strand cDNA Synthesis Kit were procured from Thermo Fisher Scientific Inc. (Waltham, MA, United States). Primary antibodies for Western blot (WB) and immunohistochemistry (IHC) included mouse or rabbit polyclonal antibodies against CYP1A2, CYP2C11, CYP3A1, and histone H3 (ProteinTech, Rosemont, IL, United States), P-gp (Abcam, Cambridge, MA, United States), CAR, PXR, and RXRα (Invitrogen, Carlsbad, CA, United States), and UGT1A1 and β-actin (Abclonal, Wuhan, China). An enhanced chemiluminescence reagent kit was obtained from Zen-bio (Shanghai, China). HRP-conjugated goat anti-rabbit and mouse IgG (H + L) secondary antibodies were sourced from Affbiotech (Jiangsu, China) and Abclonal (Wuhan, China), respectively. Fluorescein Isothiocyanate (FITC)-conjugated goat anti-rabbit IgG (H + L) and Cyanine 3 (CY3)-conjugated goat anti-mouse IgG (H + L) secondary antibodies were obtained from Servicebio Technology (Wuhan, China). Beyotime Biotechnology (Shanghai, China) supplied 4′, 6-diamidino-2-phenylindole (DAPI), antifade mountant, RIPA lysis buffer, the BCA protein assay kit, and nuclear and cytoplasmic extraction kits. Ultrapure water was produced on a Milli-Q Reference Ultrapure Water apparatus (Millipore, Bedford, United States). All other chemicals were of analytical grade and are commercially available.

### Animals

Male Sprague-Dawley rats (220 ± 20 g) were obtained from Dashuo Experimental Animal Co., Ltd. (Chengdu, China, Permission No. SYXK2019-031). The rats were housed in standard cages at the Laboratory Animal Research Center of Sichuan Animal Science Academy under controlled conditions of 22°C ± 2°C, with a light cycle from 7:30 to 19:30. Before experiments began, the rats underwent a 1-week acclimatization period, during which they were fed a regular rodent diet and freely accessible tap water. All experimental procedures strictly adhered to the ethical guidelines delineated in the National Institutes of Health Guide for the Care and Use of Laboratory Animals (US National Research Council 1996), ensuring their ethical and humane treatment. The study protocol was approved by the Ethics Committee of Sichuan Animal Science Academy (Approval No. 2022-022).

### Study design, formulation, and dosing regimen

Rats were randomly divided into two groups of 17 each: a control group (CTR) and an MB treatment group. According to the Veterinary Pharmacopoeia of the People’s Republic of China (2020 Edition), the recommended dosage of *Flos Lonicerae* for pigs is 5-10 g, equivalent to approximately 0.20 g/kg body weight (BW). After converting this dosage for rats, the estimated oral dose was determined to be approximately 0.90 g/kg BW. Our assays and reference reports (Zhao et al., 2015; Zhou et al., 2023) indicate that MB comprises approximately 5%–10% of *Flos Lonicerae*, so the calculated oral dose was 45–90 mg/kg BW. We selected an MB dose of 60 mg/kg BW for the rats in this experiment, administered intragastrically in sterile water once per day for one consecutive week. The CTR group was given an equivalent volume of sterile water via intragastric administration.

### Effects of MB on florfenicol pharmacokinetics in rats

On Day 8 of the experiment, following a 12-hour fasting period, eight rats from each group were randomly selected and given an intragastric administration of florfenicol at a 25 mg/mL concentration, corresponding to a dose of 25 mg/kg BW. Blood samples were then collected from the tail vein at predetermined intervals of 0.083, 0.25, 0.50, 0.75, 1, 2, 4, 6, 8, 10, and 12 h post-administration (*n* = 8).

The preparation and analysis of plasma florfenicol levels followed the methodology outlined in a previous study (Li et al., 2020), with minor modifications. Briefly, 50 µL of plasma was mixed in a 2 mL tube with 2.5 µg chloramphenicol (internal standard) in 5 µL methanol before adding 200 µL ethyl acetate. After vortex-mixing and centrifugation at 4,000 rpm for 10 min, the supernatant was moved to a new tube and the subnatant was re-extracted with an additional 200 µL of ethyl acetate. The combined supernatants were evaporated under nitrogen at 40°C, and the residue was dissolved in 50 µL of the mobile phase. The solution was then centrifuged at 13,000 rpm for 10 min at 4°C before injecting the supernatant into the ultra-high performance liquid chromatography (UHPLC) system for analysis.

UHPLC analyses were conducted on an UltiMate 3000 HPLC system (Thermo Fisher Scientific Inc., Chelmsford, MA, United States). A Diamonsil C18 column (4.6 mm × 250 mm, 5 μm; Welch Materials, Inc., West Haven, CT, United States) was employed to detect florfenicol and chloramphenicol simultaneously, at a constant 40°C temperature, with a UV wavelength of 223 nm. The mobile phase consisted of water and acetonitrile (73:27, v/v) at a flow rate of 1.0 mL/min, with a sample injection volume of 20 μL. This bioanalytical method had been previously validated (Li et al., 2020).

### Effects of MB on CYP450 activities using a cocktail method

A cocktail method was employed to investigate hepatic CYP450 activity. On Day 8 of the experiment, following a 12-hour fasting period, six rats were randomly selected from each group and administered an intragastric cocktail suspension at a 10 mL/kg BW dose. The suspension contained phenacetin at a concentration of 4 mg/mL (40 mg/kg BW) as a CYP1A2 probe, and tolbutamide at a concentration of 2 mg/mL (20 mg/kg BW) as a CYP2C11 probe. Blood samples were collected from each rat at time intervals of 0.167, 0.25, 0.50, 1, 2, 4, 8, 12, 24, and 36 h post-administration of the probe drugs (*n* = 6). Sample preparation and analysis of the probe drugs were performed according to methods established in our previous research (Li et al., 2017).

In brief, within a 1.5 mL centrifuge tube, 50 μL of methanol containing carbamazepine (4 μg/mL) as an internal standard was added to 50 μL of the plasma sample. The mixture was vortexed for 30 s, followed by ultrasonic treatment for 10 min. After centrifugation at 13,000 rpm for 5 min, the supernatant was passed through a 0.45 μm membrane filter and injected into the UltiMate 3000 HPLC system at a 20 μL volume for analysis. The probes and the internal standard were analyzed simultaneously on a Diamonsil C18 column (Welch Materials, Inc., West Haven, CT, United States) operating at a constant 35°C temperature. The mobile phase consisted of a 50 mM phosphate buffer (pH 3.2, 45:55, v/v) and methanol, with a flow rate of 1.0 mL/min. This bioanalytical method has been previously validated (Li et al., 2017).

### Determining mRNA and protein expression in liver samples

#### Sample collection

On Day 8 of the experiment, following a 12-hour fasting period, three rats from each group were randomly selected and euthanized in a carbon dioxide asphyxiation chamber. A section of each liver was excised and fixed in 4% paraformaldehyde for immunohistochemical analysis. The tissue was thoroughly dehydrated using a 30% sucrose solution to ensure optimal preservation. The remaining liver tissue was immediately frozen in liquid nitrogen and stored at −80°C for reverse transcription–quantitative polymerase chain reaction (RT-qPCR) and WB analyses.

#### Total mRNA and protein expression

Total mRNA and protein levels in the liver samples were quantified using RT-qPCR and WB, respectively, following previously established, slightly modified methods (Li S. C. et al., 2023).

For RT-qPCR, the amplification protocol involved denaturation at 95°C for 30°s, followed by 45 cycles of further denaturation at 95°C for 5°s, annealing at 55°C for 30°s, and extension at 72°C for 15°s, plus a final extension at 72°C for 30 s. GAPDH was used as the endogenous reference gene, with values normalized to the CTR group. Fold changes were calculated using the 2^−ΔΔCT^ method. The mRNA quantification followed the primer sequences listed in Table 1.

**TABLE 1:** The sequences of the forward and reverse primers used for RT-qPCR.

Enzymes	Forward	Reverse
CYP1A2	5′-GAATGTCACCT CAGGGAATGC-3′	5′-GACCGCCATTG TCTTTGTAGTT-3′
CYP2C11	5′-GAGGACCATTG AGGACCGTATT-3′	5′-GGAGCACAGC CCAGGATAAA-3′
CYP3A1	5′-TTCCATCTTAT GCTCTTCACCG-3′	5′-ACCTCATGCCA ATGCAGTTC-3′
UGT1A1	5′-CACGAAGTGGT GGTCATAGCA-3′	5′-TTTTGGAATGG CACAGGGTA-3′
MDR1	5′-TCCTATGCTGC TTGTTTCCG-3′	5′-AGACTTTGGCC TTCGCGTA-3′
CAR	5′-TGCCTCTGCTC ACACACTTTGC-3′	5′-GATTTCCACAG CCGCTCCCTTG-3′
PXR	5′-CCTGAAGATCA TGGCTGTCCTCAC-3′	5′-CCGTCCGTGCT GCTGAATAACTC-3′
RXRα	5′-CCTACACCTGC CGTGACAACAAG-3′	5′-TCGCTGCCGCT CCTCCTG-3′
GAPDH	5′-CAAGTTCAACG GCACAGTCAA-3′	5′-CGCCAGTAGAC TCCACGACA-3′

For WB analysis, primary antibody concentrations included CYP1A2 (1:1,000), CYP2C11 (1:2,000), CYP3A1 (1:1,000), UGT1A1 (1:2,000), P-gp (1:5,000), CAR (1:1,000), PXR (1:1,000), RXRα (1:2,000), and β-actin (1:50,000). The Tanon 5,200 chemiluminescent imaging system (Tanon Life Science Co., Ltd., Shanghai, China) was used to visualize and photograph the protein bands for semi-quantitative analysis based on band densitometry. Band densities were analyzed in ImageJ software (National Institutes of Health, Bethesda, MD, United States). To assess the nuclear and cytoplasmic expression levels of PXR, CAR, and RXRα proteins in hepatocytes, the proteins were fractionated using a commercial nuclear and cytoplasmic extraction kit following the manufacturer’s protocol. Quantitative changes in these target proteins were determined via WB as mentioned above, with β-actin and histone H3 as respective cytoplasmic and nuclear protein loading controls. The primary antibody dilutions for these analyses were CAR (1:1,000), PXR (1:1,000), RXRα (1:2,000), histone H3 (1:5,000), and β-actin (1:50,000).

#### Immunofluorescence staining

For IHC analysis, liver tissue sections were prepared at a thickness of 3 µm and mounted on APES-coated slides. The sections were deparaffinized in xylene and rehydrated with graded ethanol. They were heated in 0.01 M citrate buffer (pH 6.0) for antigen retrieval, followed by blocking of endogenous peroxidase activity with 3% (v/v) H_2_O_2_ at room temperature for 25 min. To minimize nonspecific protein binding, normal goat serum (Servicebio, Wuhan, China) was applied for 30 min at room temperature. The sections were then incubated overnight at 4°C with constant mixing, using rabbit anti-CAR and mouse anti-RXRα, each at a 1:100 dilution, as primary antibodies for one set and rabbit anti-PXR and mouse anti-RXRα, again each at a 1:100 dilution, for the other set. A negative control was prepared by using phosphate-buffered saline instead of the primary antibody.

Following primary antibody incubation, both sets of sections were incubated with secondary antibodies: CY3-conjugated goat anti-mouse IgG (1:200) and FITC-conjugated goat anti-rabbit IgG (1:100) with constant mixing for 30 min at 37°C. The sections were mounted with antifade mountant on adhesive microscope slides, then images were captured from an Olympus VS200 Research Slide Scanner (Olympus Corp., Tokyo, Japan).

### Statistical analysis

Pharmacokinetic parameters were calculated using a noncompartmental approach with Data Analysis System software from the Chinese Pharmacological Society (Beijing, China). All data are presented here as mean ± SD. A Shapiro-Wild test was used to assess normality. Statistical differences between MB and CTR groups were evaluated according to an independent-sample *t*-test in GraphPad Prism 9 (San Diego, CA, United States), where a *p*-value below 0.05 is considered statistically significant.

## Results

### Effects of MB on florfenicol pharmacokinetics

**FIGURE 2:**
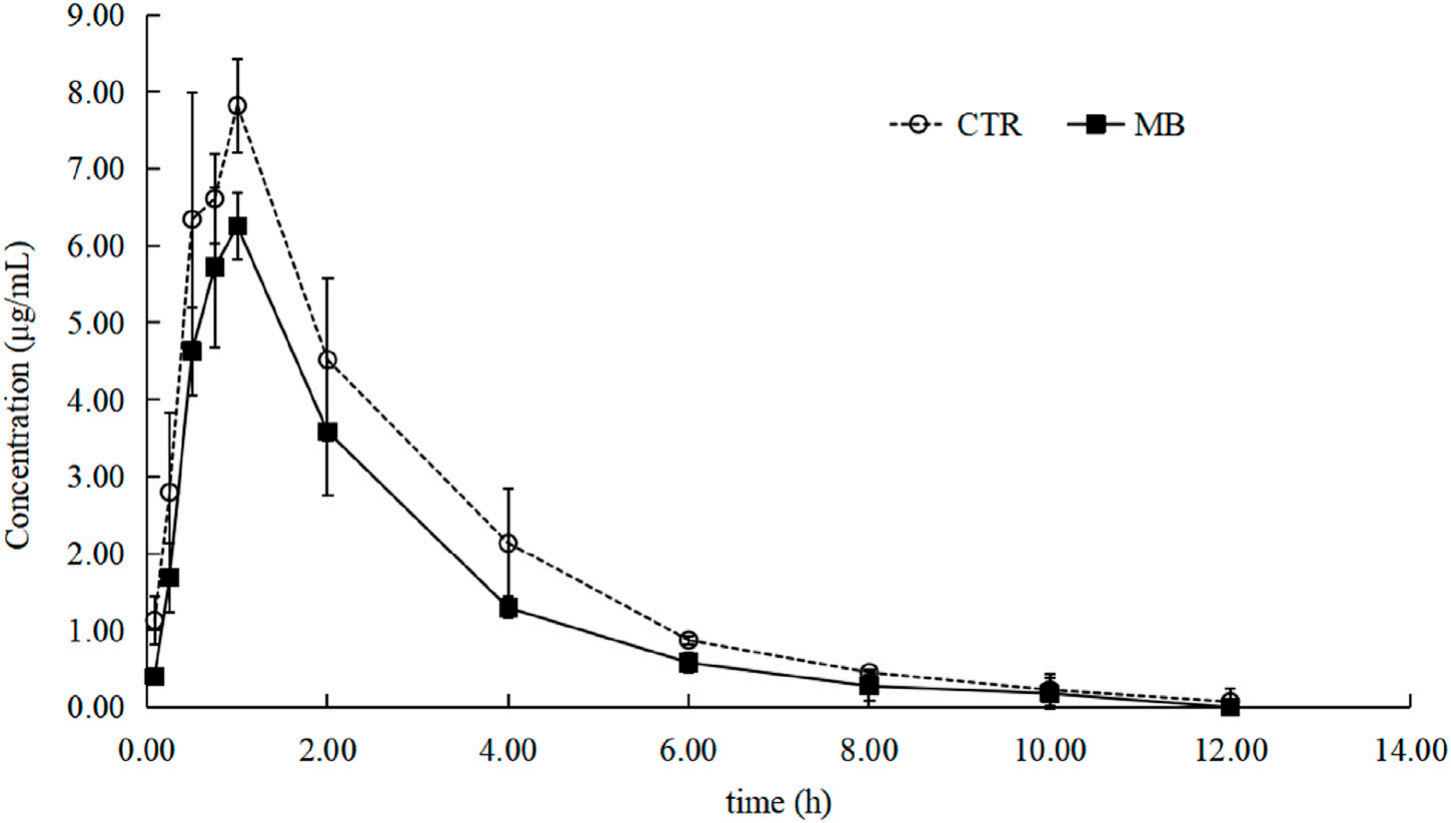
Mean plasma concentration–time profiles of florfenicol in rats after oral administration of florfenicol with or without MB pretreatment. Each symbol with a bar represents the mean value ± SD (n = 8).

The effects of MB on the pharmacokinetics of florfenicol in rats are described in Table 2 and Figure 2. After 7 days of consistent intragastric administration of MB, the area under the concentration-time curve from zero to infinity [AUC_(0-∞)_] for florfenicol in the MB group decreased to 72.04%, the mean residence time from zero to infinity [MRT_(0-∞)_] to 83.33%, the elimination half-life (t_1/2z_) to 45.26%, and the and peak concentration (C_max_) to 80.67%, all substantial reductions. Concurrently, the plasma clearance fraction of the absorbed dose (CLz/F) markedly increased, by 40.19%, in the MB group compared to the CTR group. Between the CTR and MB groups, no statistically significant differences were observed in the time to reach peak concentration (T_max_) or the apparent volume of distribution fraction of the absorbed dose (Vz/F). These changes in parameters suggest that MB may increase the absorption and decrease the elimination rate of florfenicol.

**TABLE 2:** Pharmacokinetic characteristics of florfenicol in plasma of rats after intragastric administration of florfenicol with or without MB pretreatment (n = 8, Mean ± SD).

Characteristic	CTR group	MB group
AUC_(0-∞)_ (mg/L*h)	23.50 ± 2.07	16.93 ± 2.16*
MRT_(0-∞)_ (h)	2.94 ± 0.24	2.45 ± 0.18*
t_1/2z_ (h)	1.37 ± 0.36	0.62 ± 0.02*
T_max_ (h)	0.90 ± 0.22	0.94 ± 0.13
Vz/F (L/kg)	2.22 ± 1.29	1.33 ± 0.20
CLz/F (L/h/kg)	1.07 ± 0.10	1.50 ± 0.19*
C_max_ (mg/L)	8.02 ± 0.85	6.47 ± 0.68*

*Significantly different from CTR, *P* < 0.05.

### Effects of MB on hepatic CYP1A2, CYP2C11, CYP3A1, UGT1A1, and MDR1/P-gp expression

**FIGURE 3:**
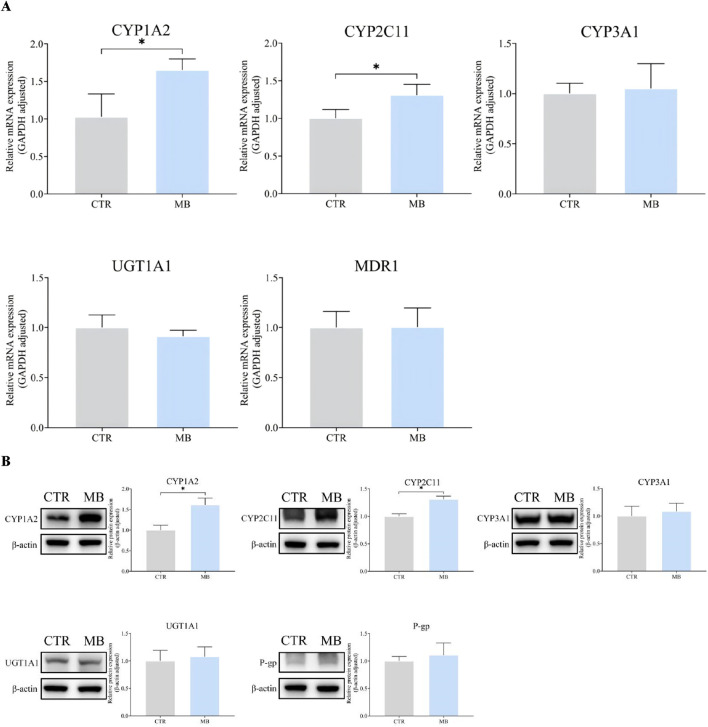
Effect of MB on the expression of CYP1A2, CYP2C11, CYP3A1, UGT1A1, and P-gp in the liver. **(A)** The relative mRNA transcript levels were analyzed by RT-qPCR analysis. **(B)** The relative protein levels were analyzed by Western blot analysis. Statistics were performed with independent-sample *t*-test (n = 3). *Significantly different from CTR rats, *P* < 0.05.

Given the significant changes observed in the pharmacokinetics of florfenicol upon co-administration with MB, we investigated the effects of MB on the expression of CYP1A2, CYP2C11, CYP3A1, UGT1A1, and MDR1/P-gp in rat livers via RT-qPCR and WB analyses. As shown in Figure 3A, we found a significant increase in the mRNA levels of CYP1A2 and CYP2C11 in the MB group after 1 week of intragastric administration, reaching 1.65-fold and 1.31-fold of the levels in the CTR group, respectively. However, we observed no statistically significant differences in the mRNA expression levels of CYP3A1, UGT1A1, and MDR1 between these groups.

Figure 3B further details the impact of MB on hepatic protein expression, including a significant increase in CYP1A2 and CYP2C11 levels in the MB group after the 7-day intragastric administration period. These protein levels reached 1.61-fold and 1.31-fold of those in the CTR group, respectively, consistent with the RT-qPCR findings. We found no statistically significant differences in CYP3A1, UGT1A1, or P-gp protein expression levels between the CTR and MB groups. These observations suggest that florfenicol pharmacokinetic variations in the rats can be attributed to the MB-induced modulation of hepatic CYP1A2 and CYP2C11 expression.

### Effects of MB on CYP1A2 and CYP2C11 activities

**FIGURE 4:**
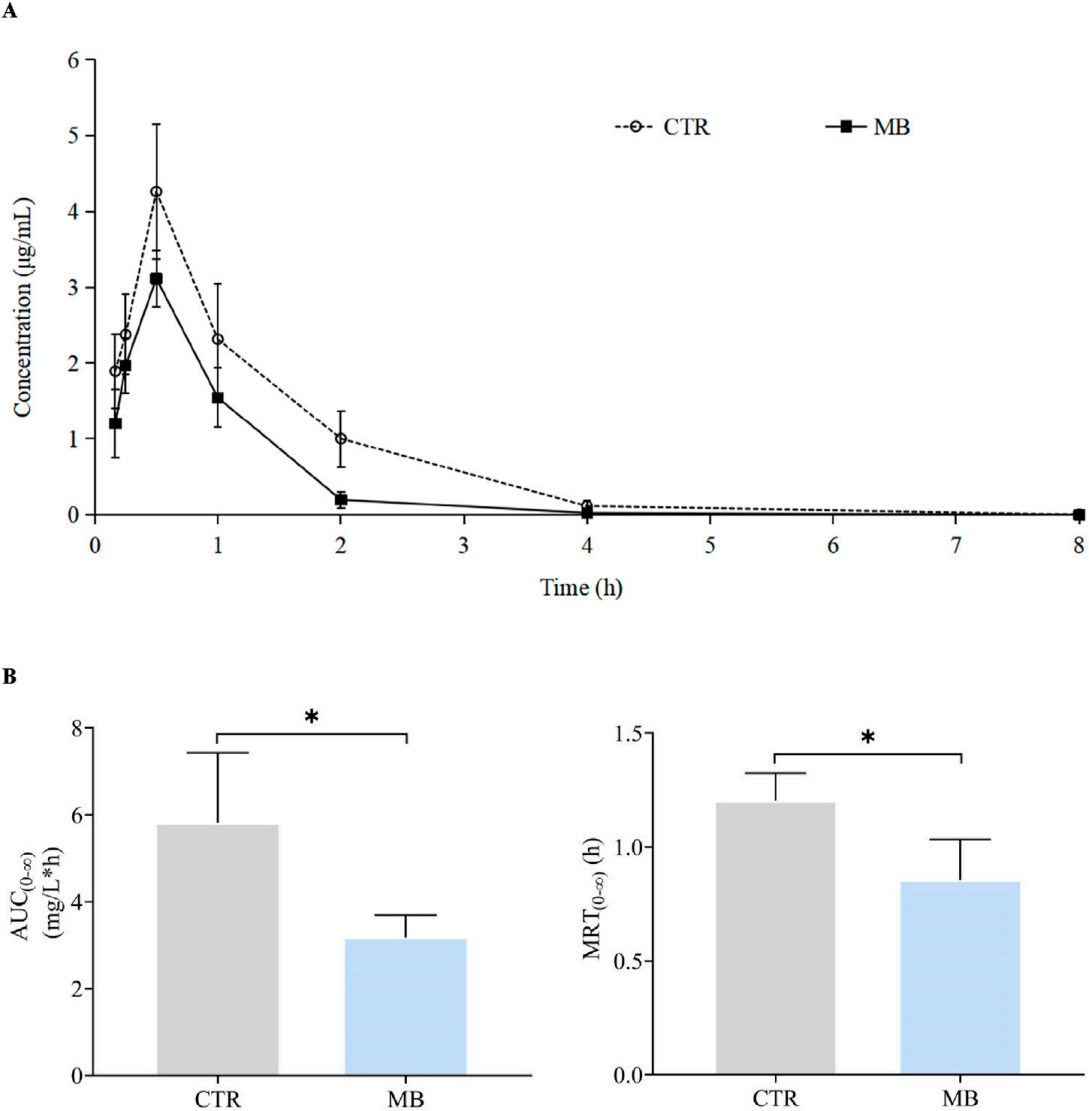
Effect of MB on the pharmacokinetics of CYP1A2 probe phenacetin in rats. **(A)** Mean plasma concentration–time profiles of phenacetin in rats after oral administration of phenacetin with or without MB pretreatment. **(B)** AUC_(0-∞)_ and MRT_(0-∞)_ of phenacetin in rats after oral administration of phenacetin with or without MB pretreatment. Statistics were performed with independent-sample *t*-test (n = 6). *Significantly different from CTR rats, *P* < 0.05.

To further clarify the effects of MB on drug-metabolizing enzyme functions, we utilized a probe drug assay (as established in our previous study) to measure the activities of CYP1A2 and CYP2C11, which had shown significant increases in expression. The impact of MB on the pharmacokinetics of phenacetin is summarized in Table 3; the mean plasma concentration-time profiles for phenacetin in both groups are illustrated in Figure 4A, and Figure 4B presents the AUC_(0-∞)_ and MRT_(0-∞)_ values for phenacetin in both groups. After 7 days of intragastric administration of MB, the AUC_(0-∞)_, MRT_(0-∞)_, and t_1/2z_ of phenacetin significantly decreased to 54.64%, 70.83%, and 74.60%, respectively, while the CLz/F of phenacetin increased by up to 73.55% compared to the CTR group.

**TABLE 3:** Pharmacokinetic characteristics of phenacetin in plasma of rats after intragastric administration of phenacetin with or without MB pretreatment (n = 6, Mean ± SD).

Characteristic	CTR group	MB group
AUC_(0-∞)_ (mg/L*h)	5.82 ± 1.61	3.18 ± 0.51*
MRT_(0-∞)_ (h)	1.20 ± 0.12	0.85 ± 0.18*
t_1/2z_ (h)	0.63 ± 0.04	0.47 ± 0.09*
T_max_ (h)	0.50	0.50
Vz/F (L/kg)	6.84 ± 2.45	8.58 ± 1.64
CLz/F (L/h/kg)	7.41 ± 2.36	12.86 ± 2.02*
C_max_ (mg/L)	4.26 ± 0.89	3.11 ± 0.38

*Significantly different from CTR, *P* < 0.05.

**FIGURE 5:**
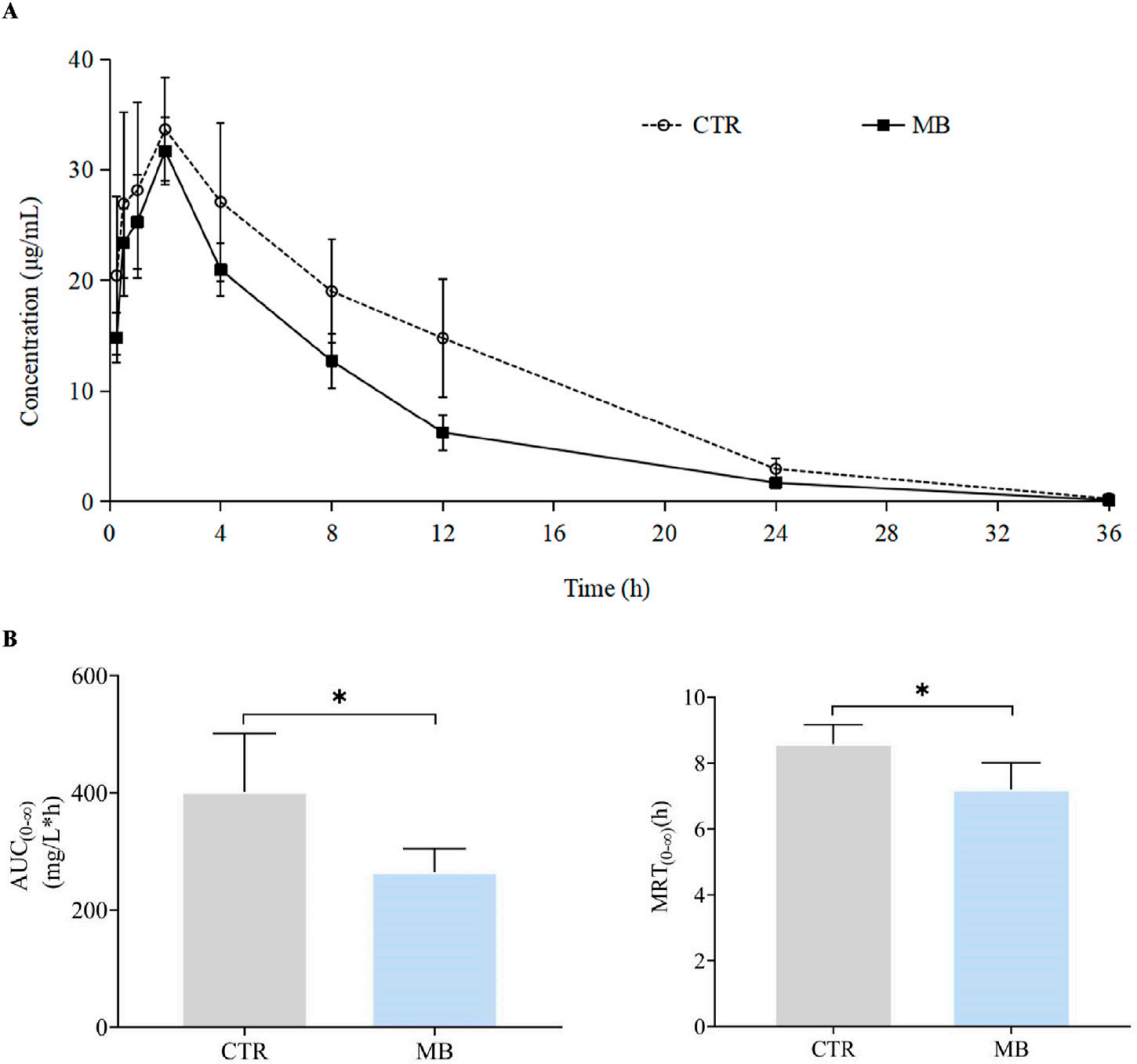
Effect of MB on the pharmacokinetics of CYP2C11 probe tolbutamide in rats. **(A)** Mean plasma concentration–time profiles of tolbutamide in rats after oral administration of phenacetin with or without MB pretreatment. **(B)** AUC_(0-∞)_ and MRT_(0-∞)_ of tolbutamide in rats after oral administration of tolbutamide with or without MB pretreatment. Statistics were performed with independent-sample *t*-test (n = 6). *Significantly different from CTR rats, *P* < 0.05.

The impact of MB on the pharmacokinetics of tolbutamide are described in Table 4, and the mean plasma concentration-time profiles of tolbutamide for the two groups are illustrated in Figure 5A; the AUC_(0-∞)_ and MRT_(0-∞)_ values for tolbutamide in both groups are depicted in Figure 5B. After intragastric administration of MB for 7 days, the AUC_(0-∞)_, MRT_(0-∞)_, and t_1/2z_ of tolbutamide decreased significantly to 65.82%, 84.01%, and 69.04%, respectively, in comparison to the CTR group. Conversely, CLz/F of tolbutamide increased significantly by 60.00%. These observations indicate that CYP1A2 and CYP2C11 activities were enhanced following 1 week of intragastric MB administration.

**TABLE 4:** Pharmacokinetic characteristics of tolbutamide in plasma of rats after intragastric administration of tolbutamide with or without MB pretreatment (n = 6, Mean ± SD).

Characteristic	CTR group	MB group
AUC_(0-∞)_ (mg/L*h)	401.68 ± 99.25	264.38 ± 44.53*
MRT_(0-∞)_ (h)	8.57 ± 0.60	7.20 ± 0.81*
t_1/2z_ (h)	4.49 ± 0.43	3.10 ± 0.68*
T_max_ (h)	1.83 ± 0.41	2.00
Vz/F (L/kg)	0.35 ± 0.11	0.33 ± 0.14
CLz/F (L/h/kg)	0.05 ± 0.01	0.08 ± 0.01*
C_max_ (mg/L)	33.65 ± 4.67	31.69 ± 3.06

*Significantly different from CTR, *P* < 0.05.

### Effects of MB on hepatic PXR, CAR, and RXRα expression

#### mRNA and total protein expression of hepatic PXR, CAR, and RXRα

**FIGURE 6:**
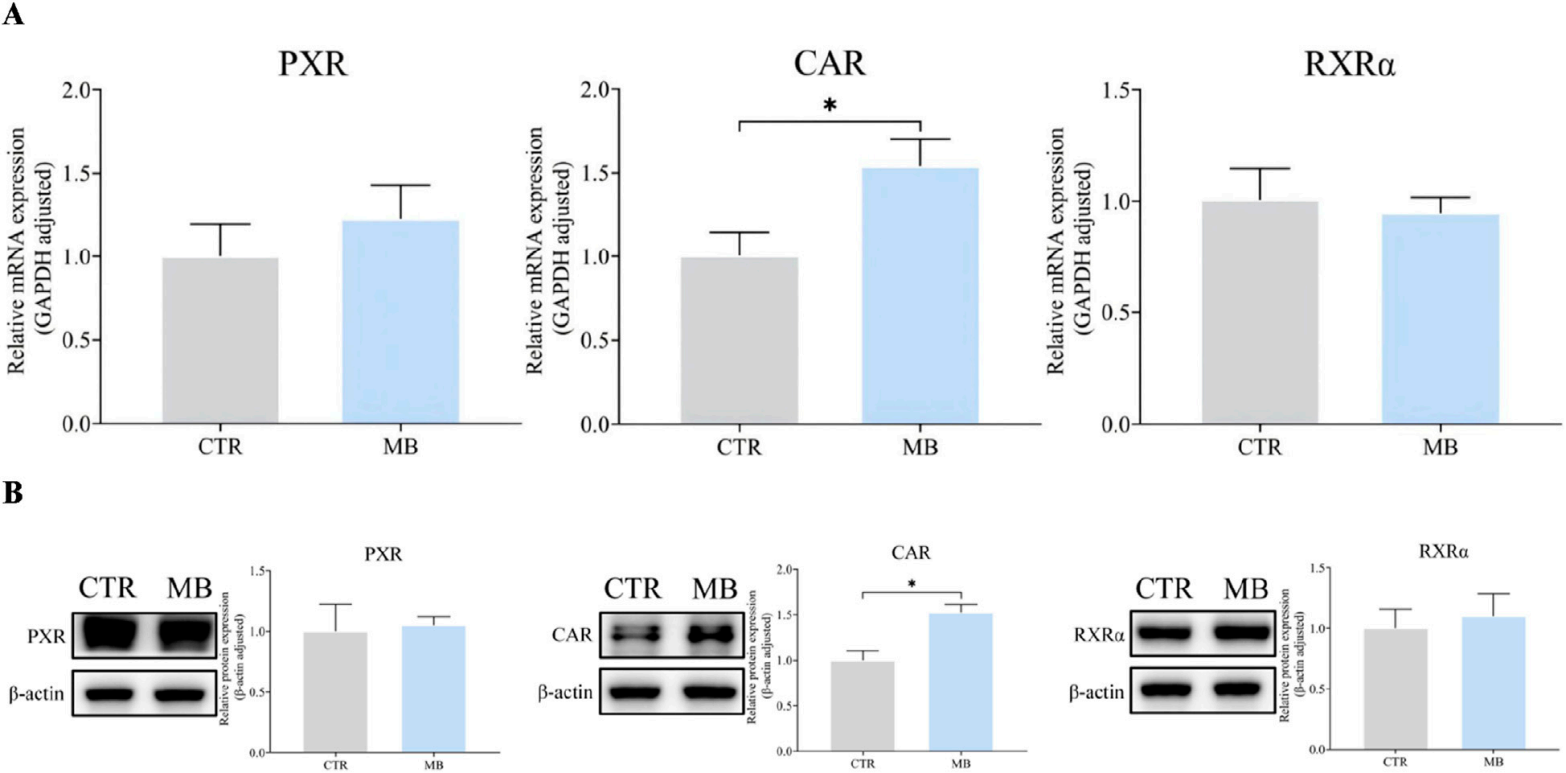
Effect of MB on the mRNA and total protein expression of CAR, PXR, and RXRα in the liver. **(A)** The relative mRNA transcript levels were analyzed by RT-qPCR analysis. **(B)** The relative protein levels were analyzed by Western blot analysis. Statistics were performed with independent-sample *t*-test (n = 3). *Significantly different from CTR rats, *P* < 0.05.

Given the observed increase in CYP1A2 and CYP2C11 expression, we hypothesized that these CYP450 expressions might be regulated by upstream transcription factors like PXR, CAR, and RXRα. To verify this, we examined the mRNA and total protein expression of these nuclear receptors in rat livers post-intragastric administration of MB. As shown in Figure 6A, we observed a significant increase in CAR mRNA levels in the MB group, reaching 1.54-fold the levels in the CTR group. However, we found no statistically significant differences in the mRNA expression of PXR and RXRα between the CTR and MB groups.

Similarly, Figure 6B shows a notable increase in CAR protein levels in the MB group, reaching 1.51-fold of those in the CTR group. Once again, there was no significant difference among the protein expression levels of PXR and RXRα between groups.

#### Nuclear and cytoplasmic protein expression of hepatic PXR, CAR, and RXRα

**FIGURE 7:**
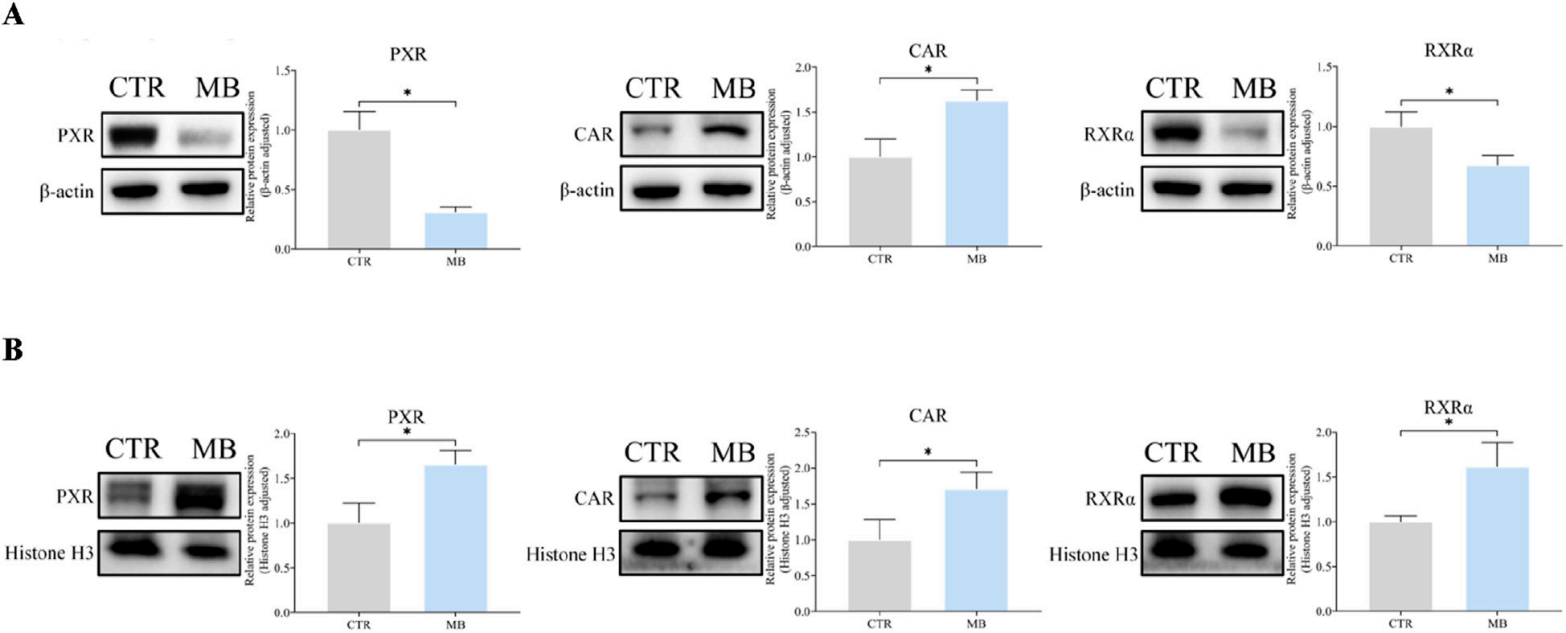
Effect of MB on the cytoplasmic and nuclear protein expression of hepatic CAR, PXR, and RXRα in the liver. **(A)** The relative cytoplasmic protein expression of hepatic CAR, PXR, and RXRα were analyzed by Western blot analysis. **(B)** The relative nuclear protein expression of hepatic CAR, PXR, and RXRα were analyzed byWestern blot analysis. Statistics were performed with independent-sample *t*-test (n = 3). *Significantly different from CTR rats, *P* < 0.05.

Figures 7A, B reflect our detailed analysis of the impact of MB on hepatic PXR, CAR, and RXRα cytoplasmic and nuclear protein expressions. CAR protein expression was significantly elevated in both cytoplasmic and nuclear fractions in the MB group, reaching 1.63-fold and 1.71-fold, respectively, of the levels in the CTR group. PXR protein expression demonstrated a contrasting pattern: cytoplasmic PXR protein levels significantly decreased to 0.31-fold of those in the CTR group, while nuclear PXR protein levels increased by 1.65-fold compared to the CTR group. Similarly, RXRα protein expression showed divergent patterns: cytoplasmic RXRα protein levels significantly decreased to 0.68-fold of those in the CTR group, while nuclear RXRα protein levels increased by 1.62-fold compared to the CTR group.

**FIGURE 8:**
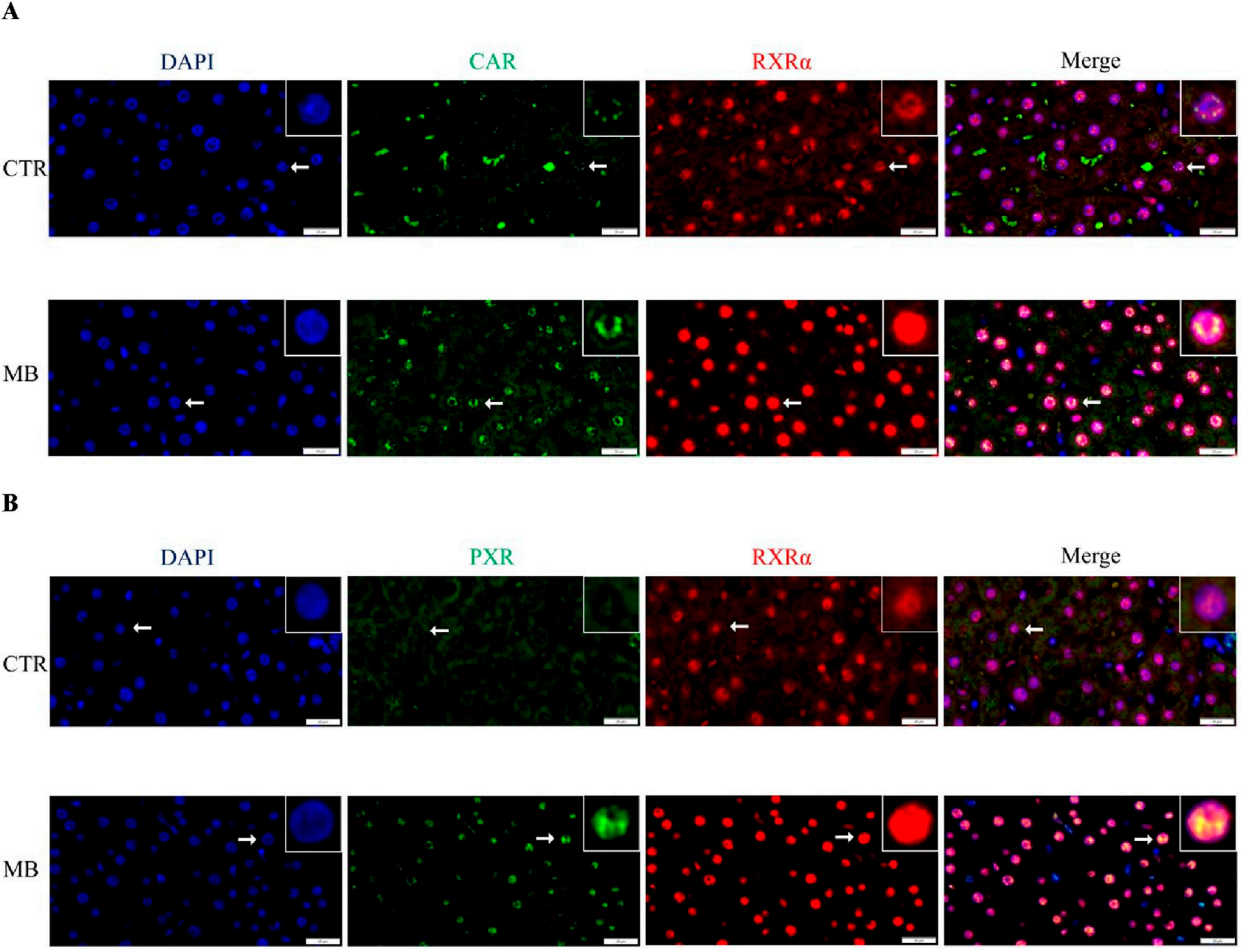
Double immunofluorescence staining demonstrates the nuclear colocalization of hepatic CAR/RXRα **(A)** and PXR/RXRα **(B)** in hepatocyte nuclei (n = 3). CAR and PXR are shown in green, RXRα in red, and nuclei are stained with DAPI (blue). Scale bar: 20 μm.

Double immunofluorescence staining was performed to further explore the localization and expression of CAR/RXRα and PXR/RXRα in hepatocytes. In the CAR/RXRα staining (Figure 8A), CAR expression was markedly increased in both the cytoplasm and nucleus of hepatocytes in the MB group over the CTR group. Additionally, nuclear RXRα expression showed a notable increase, while cytoplasmic RXRα expression decreased significantly in the MB group. Similarly, in the PXR/RXRα staining (Figure 8B), both PXR and RXRα expression in the MB group increased substantially in the nucleus, accompanied by a significant reduction in their cytoplasmic expression in hepatocytes.

## Discussion

In the initial phase of this study, we administered MB intragastrically to rats for one consecutive week followed by a single intragastric dose of florfenicol on the eighth day. This led to significant alterations in the pharmacokinetics of florfenicol. Specifically, the MB group exhibited a notable decrease in the C_max_ of florfenicol, alongside a significant increase in CLz/F and corresponding shortening of t_1/2z_, compared to the CTR group. These observations suggest that co-administration of MB may inhibit florfenicol absorption and accelerate its elimination. We infer accordingly that these pharmacokinetic changes are primary factors contributing to the significant decrease in florfenicol’s AUC_(0-∞)_ and MRT_(0-∞)_ in the MB group, leading to reduced plasma concentrations of the drug.

To investigate the mechanism by which MB accelerates the metabolism of florfenicol, we investigated its impact on the expression of rat liver Phase I enzymes CYP1A2, CYP2C11, and CYP3A1, Phase II enzyme UGT1A1, and efflux transporter P-gp at both transcriptional and protein levels. Our findings revealed that a 1-week continuous intragastric administration of MB significantly increased the transcriptional and protein expression levels of hepatic CYP1A2 and CYP2C11. Using the cocktail method, widely used to assess CYP450 enzyme activity levels (Frye et al., 1997; de Andrés and LLerena, 2016), we found that MB significantly induced the activities of CYP1A2 and CYP2C11. Specifically, phenacetin and tolbutamide were used as probe drugs for CYP1A2 and CYP2C11, respectively, with their AUC_(0-∞)_ and MRT_(0-∞)_ measured according to a previously established UHPLC approach (Li et al., 2017). Co-administration of MB significantly decreased both phenacetin and tolbutamide’s AUC_(0-∞)_ and MRT_(0-∞)_, indicating that MB functions as an inducer of CYP1A2 and CYP2C11 activities. Notably, the AUC_(0-∞)_ of phenacetin and tolbutamide in the MB group were 54.64% and 65.82% of those in the CTR group, meeting the FDA’s criteria for moderate inducer (Food and Drug Administration, 2020).

Florfenicol, a widely used veterinary antibiotic, has partially elucidated metabolic pathways in rodents; CYP3A/P-gp and CYP1A2 have been identified as key factors in rabbits and rats, respectively (Liu, 2011; Liu et al., 2012). Based on our findings, the increased expression and enzymatic activity of CYP1A2 induced by MB may contribute to this acceleration in florfenicol metabolism. Although CYP2C11 induction suggests a potential link to increased florfenicol metabolism, this association remains speculative and requires further investigation.

Previous studies have shown that both CAR and PXR can form heterodimeric complexes with RXRα, which translocate to the nucleus of hepatocytes to initiate the transcription of target genes (Honkakoski and Negishi, 1997; Honkakoski et al., 1998; Yan et al., 2015; Rakateli et al., 2023). In this study, CAR was significantly upregulated in both the cytoplasm and nucleus post-MB administration compared to controls, resulting in a significant increase in total CAR protein. Interestingly, while total PXR and RXRα protein expressions in hepatocytes did not differ significantly between MB and CTR groups, we did find a significant decrease in cytoplasmic expression and a corresponding increase in nuclear expression for both proteins in the MB group, similar to the pattern observed for CAR in cell nuclei. This suggests that MB may induce the nuclear translocation of both PXR and RXRα, potentially shifting to the nucleus from the cytoplasm. Double immunofluorescence colocalization staining analysis of PXR/RXRα and CAR/RXRα yielded similar results, showing increased colocalization of both CAR/RXRα and PXR/RXRα proteins in hepatocyte nuclei in the MB group over the CTR group. These preliminary observations suggest that MB induces nuclear colocalization of both CAR/RXRα and PXR/RXRα, potentially triggering the transcription of inducible genes like those encoding CYP450 enzymes.

In the regulation of CYP450 enzymes, PXR controls the expression of genes such as CYP1A2, 2A6, 2B, 2C, and 3A, while CAR regulates CYP1A, 2A6, 2B, 2C8, 2C9, and 3A4 (Sueyoshi et al., 1999; Ferguson et al., 2002; Willson and Kliewer, 2002; Wang et al., 2003; Itoh et al., 2006; Al-Dosari et al., 2006; Pelkonen et al., 2008; Congiu et al., 2009; Yoshinari et al., 2010; Tojima et al., 2012; Manda et al., 2017; Manikandan and Nagini, 2018; Duan et al., 2020). Ferguson et al. (2002) highlighted CAR’s role in regulating CYP2C9 transcription after finding a CAR/PXR binding site within the promoter region of the CYP2C9 gene. Similarly, Al-Dosari et al. (2006) identified the element responsible for PXR- and CAR-mediated activation within the −2,000 to −1,000 bp region of the CYP2C9 5′-flanking sequence, further elucidating the role of PXR and CAR as key transcription factors in CYP2C9 expression. Given the homology between rat CYP2C11 and human CYP2C9, it is likely that the observed upregulation of CYP2C11 in our MB group resulted from MB-mediated modulation of CAR and PXR. Regarding CAR and PXR regulation of CYP1A genes, Yoshinari et al. (2010) demonstrated that CAR drives xenobiotic-induced expression of CYP1A1 and CYP1A2 independently of the aryl hydrocarbon receptor (AhR), acting through the cis-regulatory element ER8. Additionally, Manda et al. (2017) showed that Kratom (*Mitragyna speciosa*) extracts and alkaloids significantly activate PXR, resulting in increased CYP1A2 expression and activity. Collectively, these findings support our finding that MB-induced nuclear translocation of CAR/PXR may contribute to CYP1A2 upregulation. However, given AhR’s recognized role as a key regulator of CYP1 genes (Ciolino et al., 1998; Beedanagari et al., 2009), further research is needed to investigate MB’s effects on drug metabolism via the AhR pathway. Furthermore, we also found no significant changes in the expression of UGT1A1, P-gp and CYP3A1 in the MB group. While these are known to be regulated by PXR and CAR (Willson and Kliewer, 2002; Zhou et al., 2005), the absence of significant changes may suggest the involvement of additional regulatory pathways, such as AhR and peroxisome proliferator-activated receptor, which could influence the expression of these enzymes and transporters (Wallace and Redinbo, 2013).

Although specific investigations regarding MB’s impact on drug-metabolizing enzymes and transporters are limited, MB’s classification as a triterpenoid allows us to draw valuable insights from earlier studies on triterpenoids, despite variations in results due to differences in dosage, administration period, structural diversity, and other factors. For example, Song et al. (2024) demonstrated that oleanolic acid, a pentacyclic triterpenoid, increases nuclear accumulation of PXR and induces the expression of PXR downstream proteins like CYP3A11, UGT1A1, and Glutathione S-Transferase Mu 2, as well as in AML12 and HepRG cells, in mice. Lu et al. (2017) found that cucurbitacin E, a tetracyclic triterpenoid isolated from Cucurbitaceae, induces CYP3A and P-gp in rats after long-term treatment though it inhibits CYP3A and P-gp activities after acute dosing. Additionally, Vaghela et al. (2018) observed substantial inhibition of various CYP activities by triterpenoid saponins in *Gymnema sylvestre R. Br.*, an Indian medicinal herb. This body of evidence significantly contributes to our understanding of MB’s potential influence on drug-metabolizing enzymes, transporters, and drug metabolism. This study specifically explored the effects of MB on the expression of major nuclear receptors, efflux transporters, and Phase I and Phase II metabolic enzymes. These findings may lay a workable foundation for future scientific inquiries into the complex mechanisms governing MB’s influence on drug metabolism.

This work was not without limitations. Firstly, species differences raise questions regarding specificity. For example, the ligand-binding domain of porcine and rat PXR shares only 76% sequence identity (Moore et al., 2002), which may explain the substantial species-specific differences in nuclear receptor regulation of target genes. Thus, porcine or transgenic rodent models may provide more relevant insights into pig-specific drug responses related to drug metabolism. Additionally, we focused solely on the effects of MB on CAR/PXR-CYP450 expression and florfenicol pharmacokinetics in healthy rats, without establishing a bacterial infection model. Further, supplementary molecular experiments (e.g., CAR siRNA assays) are needed to confirm the mechanism by which MB regulates CYP450 via CAR/RXRα or PXR/RXRα. Lastly, while this study primarily examined the effects of MB on hepatic drug-metabolizing enzymes and transporters, we did not address factors influencing florfenicol absorption in the small intestine. We plan to systematically investigate these factors in the next phase of our research.

In summary, the findings of this study suggest that MB significantly alters the pharmacokinetics of florfenicol, with accelerated elimination as a key effect. This phenomenon is likely driven, in part, by substantial increases in the expression and catalytic activity of CYP1A2 and CYP2C11 enzymes. Additionally, nuclear receptors CAR and PXR may contribute to the upregulation of these enzymes. These insights may provide a useful foundation for further investigating the interactions between *Flos Lonicerae* and florfenicol, contributing to a more comprehensive understanding of their combined effects.

## Data Availability

The original contributions presented in the study are included in the article/supplementary material, further inquiries can be directed to the corresponding author.
